# Metabolic Changes in Pediatric HIV-Positive Patients and Potential Lifestyle Interventional Strategies

**DOI:** 10.7759/cureus.14556

**Published:** 2021-04-19

**Authors:** Falguni Patel, Christina Kennedy

**Affiliations:** 1 Internal Medicine/Pediatrics, Alabama College of Osteopathic Medicine, Dothan, USA; 2 Physiology, Alabama College of Osteopathic Medicine, Dothan, USA

**Keywords:** pediatric hiv infection, adolescent nutrition, lifestyle intervention

## Abstract

Metabolic changes in the HIV population have been well-studied, particularly after the advent of antiretroviral therapy. More notably, the emergence of the metabolic syndrome within the HIV population, due to prolonged survival, has led to an increasing rate of cardiovascular occurrence and mortality within the population in adult life. Importance of early intervention in HIV children, particularly lifestyle modifications, is necessary to reduce cardiovascular disease (CVD) risk and mortality in adulthood. Potential clinical interventions include routine anthropometric measurements as a measure of CVD risk, a low saturated fat and high fiber diet, and vigorous aerobic exercise have been shown to decrease CVD risk in the HIV population. The literature review found multiple knowledge gaps due to minimal studies completed on the HIV population and even less on HIV-positive children. Overall, a standardized protocol was required to better care for HIV-positive children and potential future CVD mortality.

## Introduction and background

Since the 1980s, the prevalence and mortality of HIV have been intensely studied to reduce the mortality rate as the disease spreads globally. With the advent of antiretroviral therapy (ART), HIV has changed from a deadly disease to a chronic disease with more infected people surviving long-term [1,2]. This increase in survival has shed light on chronic comorbidities and the long-term effects that carry to adulthood. Metabolic changes in HIV patients have been well-studied and reported over the years. Many studies have found that the cardiometabolic effects are multifactorial, with HIV, ART, and poor lifestyle habits playing major roles [1-3]. More notably, protease inhibitors are the more common class of ART to cause these metabolic changes and when coupled with the pro-inflammatory and immune dysregulation state caused by the disease, HIV patients are at higher rates of cardiometabolic changes and their sequelae. In fact, non-AIDS-related comorbid diseases such as cardiovascular disease and liver disease are currently the leading cause of death in HIV patients [4].

Simply put, the cardiometabolic modifications in HIV and ART receiving patients are typically regarded as a constellation of symptoms termed metabolic syndrome (MS). MS includes three or more of the five following criteria: visceral or abdominal obesity, elevated triglycerides, elevated blood pressure, increased fasting glucose, or decreased high-density lipoproteins (HDL). While the overall prevalence of MS is increasing within the general population of the United States, there is a high prevalence within the HIV population [2]. Additionally, dyslipidemia is commonly seen in HIV-infected patients; with low HDL and high triglycerides (TGs), these laboratory trends are more prevalent in ART-experienced patients as opposed to those who are ART-naive HIV patients [5]. Insulin resistance is also increased individually in HIV patients with one study showing that 40% of the population had one lipid abnormality or insulin resistance despite correcting for diet and exercise [6]. Subsequently, it appears that obesity is one of the more important metabolic changes seen in HIV patients, particularly high visceral fat accumulation as it can be a great indicator of cardiovascular disease later in life [7]. Whether these metabolic changes occur alone or as MS, they all have an increased risk of cardiovascular disease later in life with MS being the most powerful predictor [2]. 

The goal of this review is to assess potential early interventions via lifestyle modification and early identification of high-risk factors that can be implemented in pediatric HIV patients to reduce the long-term sequelae of the metabolic changes. 

## Review

Methods

A literature review was conducted using PubMed with keywords including “physical activity in HIV patients,” “nutrition and HIV,” “nutritional deficiencies in HIV,” “lifestyle interventions in HIV,” and “anthropometric measurement and HIV.” Studies were limited within the last 10 years from 2010 to 2020 with no restriction on the age of participants, type of study or literature, or location of study. Literature that was not directly related to cardiometabolic changes in HIV was excluded, as were the studies that were not readily accessible without a paid subscription.

Interventions

The long-term sequelae of MS and other HIV-related cardiometabolic changes are particularly important to consider in HIV-affected children. As Innes et al. mentioned, the effects of ART on lipids and insulin resistance tend to accumulate over time and therefore it is important to monitor and intervene early in life to delay the long-term effects [6]. More notably, increased rates of atherosclerosis development and nonalcoholic fatty liver disease development can contribute greatly to non-AIDS-related mortality later in life [3,8]. As numerous studies have mentioned, the metabolic changes are multifactorial encompassing disease state, ART class, genetics, diet, and physical activity [9,10]. Therefore, this review will limit interventions to modifiable lifestyle modifications by addressing deficiencies specific to the HIV population.

Nutritional Strategies

It is well-documented that a healthy, balanced diet can positively impact cardiometabolic changes in both the general and HIV-positive populations [11-13]. Starting nutritional intervention early in life can endorse healthy habits into adulthood and prevent cardiometabolic adverse effects. This is particularly true for the pediatric HIV population as early intervention can delay or reduce cardiovascular incidents and mortality in adulthood [14]. As a part of the Pediatric European Network for Treatment of AIDS Guidelines, it has been reported that clinicians disclose HIV diagnosis earlier than prior years with a focus on healthy diet and lifestyle starting in childhood [14]. Specifically, disclosing diagnosis prior to adolescence is beneficial, although it is varied upon on maturity of the patient and approval of the family [14].

Diet

To provide personalized lifestyle modification suggestions, it is important to understand the deficiencies pertaining to the HIV-positive population. The Healthy Eating Index measures overall diet quality and adherence to the Dietary Guidelines for Americans and it can be used to find deficiencies in nutritional intake [15]. Weiss et al. found that the HIV population has a lower Healthy Eating Index (HEI) overall compared to the general population, typically with more saturated fats and empty caloric intake and decreased healthy fats and plant protein [15]. The American Dietetic Association has seen similar findings of the HIV population tending to have higher saturated fats and low fiber in their diets [11]. These potential deficiencies can be detected with a three-day food record, which has been shown to be more beneficial than the typical food frequency questionnaires [11]. Important aspects to note when suggesting nutritional care to the HIV-positive population is that prescribing a specific diet tailored to the patient's diet record is reported to be the best intervention [16]. Moreover, altering a single factor at a time proves to be more effective than suggesting multiple changes simultaneously. For instance, Webel et al. found that the HIV population was more likely to alter sugar beverages and overall carbohydrates first, thus making it a potential starting point in advocating for healthy diets [12]. Furthermore, barriers to a healthy diet must be considered on an individual basis. Typical barriers include the cost of the diet, time to prepare the foods, and lack of housing while those particular to the HIV population include appetite changes due to ART as well as depression and psychiatric illnesses [13]. Factors associated with diet adherence include access to medical professionals giving diet-specific advice, using personal motivators such as weight loss and appearance, and having cost-effective options [17]. 

When considering a preferable prescription diet for HIV-positive patients, Anjos et al. found that a low glycemic index diet coupled with a low saturated fat intake and high intake of vitamins, fibers, and unsaturated fats can help reduce triglycerides and low-density lipoprotein (LDL) cholesterol in the HIV population with cardiovascular risk factors [18]. One study found that following the Mediterranean diet improved HDL levels, healthy cholesterol, and lower insulin resistance in the HIV population [17]. The Mediterranean diet is a well-researched diet focusing on vegetables and fruits as well as limiting refined sugar intake. Such a diet has been shown to improve metabolic syndrome sequelae, more notably cardiovascular disease in the general population, particularly when implemented early [19].

Micronutrient Supplementation

Supplementation to diet was also investigated in the review. Omega-3 supplementation has been shown to reduce triglycerides in the HIV population while also raising high-density lipoprotein (HDL) [16]. The same study found that chrome nicotinate did not help to improve dyslipidemia, though they did see a reduction in total body fat when compared to baseline [16]. While the effects of omega-3 supplementation are well-researched in the general population in reducing lipids, supporting its use as adjunctive treatment with diet and exercise, their long-term effects on the HIV population and those on ART are widely unknown. Moreover, vitamin D deficiency is found in a majority of the HIV population with various causes, including ART exposure, chronic inflammatory state as well as HIV proteins directly impacting enzymes involved in vitamin D production and activation [20]. One study found that 44.6% of their HIV participants had vitamin D deficiency [21], while another study reports that the numbers vary from 24% to 72% depending on climate, age, and geographic area [20]. There have been observational studies that show an association between low vitamin D levels and increased cardiovascular disease [22]. Further investigations show an inverse relationship between carotid media-intima thickness (CMIT), a measure of subclinical vascular disease, and vitamin D levels in HIV patients [22]. There was also an association with low vitamin D levels and subclinical atherosclerosis when compared to HIV patients without deficient vitamin D levels [21]. However, despite multiple observational studies indicating a relationship between vitamin D levels and CMIT, atherosclerosis, and coronary artery calcification, randomized control trials are limited as to whether supplementation can reduce cardiovascular events. The study found that oral vitamin D supplementation did not improve endothelial function that was measured with flow-mediated dilation, which is a marker for cardiovascular risk, in a vitamin D deficient HIV-positive population [23]. However, a limitation of this study was that participants did not reach sufficient serum vitamin D levels due to very low baseline concentrations. Thus, further investigations are warranted as a dietary adjunct in HIV-positive patients.

Physical Activity 

In the general population, physical activity has also proven to delay CV sequelae from metabolic abnormalities especially when coupled with the dietary intervention [11,14]. When applying physical activity to the HIV-positive pediatric population, it is once again important to determine the unique challenges that face them. In general, the HIV population has been found to have not only lower self-reported activity levels, but those who did report were more likely to mention light activity levels [13]. When looking specifically at children with HIV, the majority of children preferred low-impact exercise such as soccer and walking in boys and jogging and walking in girls [24]. When further analyzing the data, the study found that the pediatric participants did not partake in activity over the weekends and most of the activity was completed in physical education classes throughout the week at school [24]. Group-prescribed exercise can potentially be beneficial in children as opposed to individual exercise routines. The lack of physical activity in the pediatrics HIV population is particularly concerning since early implementation promotes healthy habits as adults. In a study with HIV-positive women, those that reported vigorous activity did demonstrate cardiorespiratory fitness improvements [13]. This signifies that the type of exercise, intensity, and duration of physical activity all play a role in reducing the impact of HIV-associated metabolic changes. General recommendations include a minimum of 20 minutes of vigorous aerobic activity thrice weekly can reduce body fat, improve heart health, and improve lean body mass [24-26]. 

Structured exercise programs have been shown to be viable and improve lean body mass and cardiorespiratory fitness in a study with HIV-positive children and adolescents. Among those that returned for a follow-up after completing three months of unsupervised home-based physical activity, the majority of participants retained the benefits of the structured hospital-based exercise program even when at home [25]. Overall, reducing body fat, more notably truncal fat in the HIV population, can potentially reduce the cardiometabolic adverse effects of HIV.

Anthropometric Measurements 

Through the review, anthropometric measurements were emphasized in order to determine those with increased risk for HIV-associated cardiovascular sequelae [27,28]. The loss of peripheral fat and accumulation of truncal fat is well-reported in HIV and ART patients and can signify an increased risk of cardiovascular events in adulthood [27,28]. These measurements and a series of skin-fold measurements offer a cost-effective and quick method to identify those at risk early on for implementation of lifestyle modifications.

When using body measurements to indicate higher-risk individuals, the body mass index (BMI) has been historically used; however, the HIV population warrants special considerations [29]. BMI is correlated with increased cardiovascular events, but it is incomplete in HIV patients as it does not measure truncal fat. Lipodystrophy in HIV patients includes wasting of peripheral fat and accumulation of truncal fat, a large indicator of cardiovascular risk. In a study comparing measurements in HIV-infected versus non-infected populations, the BMIs were similar for both but the HIV population had a larger waist circumference. Simply put, BMI does not provide information on fat distribution typically seen in HIV patients [29,30].

Waist circumference (WC) is a commonly used anthropometric measurement to quantify truncal fat and is strongly associated with cardiometabolic risks in HIV-positive and uninfected populations [28,31]. The typical location for measurement of WC is midway between the iliac crest and the last rib though there are slight variations [31]. One variant of this is the waist-to-height ratio which helps differentiate between truncal versus total fat and it is also a powerful determinant of body fat in children [31]. In HIV-positive children, the abdominal skinfold (SFab) was the most indicative of fat percentage when compared to the dual x-ray absorptiometry (DXA) fat scan and WC [27,28]. These measurements of visceral adipose tissue (deeper stores of truncal fat) can indicate a child with a higher risk of insulin resistance, dyslipidemia, systemic inflammation, atherosclerosis, and other cardiovascular disease markers [28]. While it is very important to monitor these values in HIV children, it is equally important to also get extremity skin-fold measurements to get a complete picture of patient health. 

A newer cardiovascular risk marker is the lipid accumulation product (LAP), which is based on WC measurements and triglyceride levels. In the first study regarding the marker, LAP and the homeostatic model assessment for insulin resistance (HOMA-IR) were positively correlated. HOMA-IR and glucose levels were higher in the HIV-infected population compared to the control, indicating a higher risk of cardiovascular events and even insulin resistance [30].

## Conclusions

With increasing obesity and its associated cardiometabolic effects, it is important to investigate the importance of changing lifestyles early in life. This is particularly true for HIV-positive children who live a longer life and develop fatal cardiometabolic sequelae in adulthood. Implementing positive changes in childhood will improve quality of life and reduce the health costs of chronic conditions. The review identified specific challenges to lifestyle modifications that HIV-positive patients may face, such as nutritional deficiencies, barriers to implementing changes, and potential interventions to reduce risk. Early detection of cardiovascular risk is imperative through truncal fat measurements and extremity skin-fold measurements. Also, the HIV population seems to consume more saturated fats, empty calories, with diets low in fiber and unsaturated fats, and vitamin D deficiencies all of which contribute to cardiometabolic risks. Deficiencies in vigorous physical activity well as potential benefits of group exercise programs were also identified in the HIV-positive population. While these can improve cardiometabolic sequelae, it is necessary to keep in mind that lifestyle modifications are multifactorial and must be approached on an individual basis keeping in mind the patient’s specific challenges.

While there are many lifestyle interventions to reduce cardiometabolic events in the general population, there is little involving the HIV population and much less with HIV-infected children. Examining metabolic changes and lifestyle interventions in HIV children is a necessary avenue to pursue given the new challenges facing HIV-positive adults. Additionally, there is a lack of structural guidance for clinicians who treat HIV-positive children. This is understandable given the multifactorial nature of the topic, but more longitudinal studies on adolescents into adulthood and lifestyle interventions can provide further insight into cardiometabolic sequelae that will plague HIV-positive adults.
